# Measuring oxytocin release in response to gavage: Computational modelling and assay validation

**DOI:** 10.1111/jne.13303

**Published:** 2023-06-14

**Authors:** Shereen Hassan, Hala El Baradey, Mohamed Madi, Mohamed Shebl, Gareth Leng, Maja Lozic, Mike Ludwig, John Menzies, Duncan MacGregor

**Affiliations:** ^1^ Centre for Discovery Brain Sciences University of Edinburgh Edinburgh UK; ^2^ Department of Medical Physiology, Faculty of Medicine Tanta University Tanta Egypt; ^3^ Centre for Neuroendocrinology, Faculty of Health Sciences University of Pretoria Pretoria South Africa

**Keywords:** appetite, computational modelling, hypothalamus, immunoassay, supraoptic nucleus

## Abstract

In the present experiments, we tested the conclusion from previous electrophysiological experiments that gavage of sweet food and systemically applied insulin both stimulate oxytocin secretion. To do so, we measured oxytocin secretion from urethane‐anaesthetised male rats, and demonstrated a significant increase in secretion in response to gavage of sweetened condensed milk but not isocaloric cream, and a significant increase in response to intravenous injection of insulin. We compared the measurements made in response to sweetened condensed milk with the predictions from a computational model, which we used to predict plasma concentrations of oxytocin from the published electrophysiological responses of oxytocin cells. The prediction from the computational model was very closely aligned to the levels of oxytocin measured in rats in response to gavage.

## Graphical abstract

An European study of liver metastases with CT in 14 expert centers in NET treatments has shown that a ≥10% decrease in size of three liver metastases could be a more clinically relevant criterion than the current 30% threshold utilized by RECIST 1.1 for the evaluation of treatment efficacy in patients with advanced NETs. Its implementation in clinical trials is mandatory for prospective validation. Criteria based on changes in liver metastases CT‐density were not predictive of treatment efficacy.

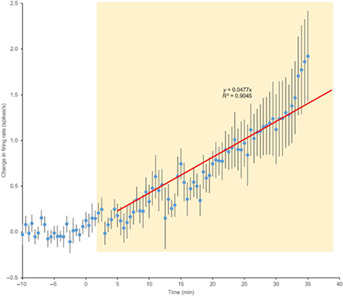

## 1 INTRODUCTION

In mammals, oxytocin is essential for mediating milk let‐down in lactation and it regulates parturition by its actions at the uterus, but the magnocellular neurones of the hypothalamus that secrete oxytocin into the blood also release oxytocin within the brain. There, oxytocin influences diverse behaviours: it promotes sexual arousal in males and sexual receptivity in females; it reduces fear and anxiety, thereby facilitating the social contact that is a prerequisite for reproduction; and it initiates maternal behaviour and affiliative behaviours between sexual partners in various species. This seems to be a coherent set of actions in support of reproductive efficacy,^1^ but oxytocin also has other, very different actions. Amongst these, it inhibits food intake: in the dorsal vagal complex of the caudal brainstem, oxytocin released from the endings of parvocellular oxytocin neurones controls gastric reflexes, including the closure of the gastric sphincter in response to gastric distension. The magnocellular oxytocin neurones are also activated during feeding, and can release oxytocin within the brain from their dendrites and from axon collaterals.^2^

The pathways and mechanisms by which oxytocin neurones are activated during feeding remain to be fully defined. The vagus nerve is one route; cholecystokinin released from the gut can stimulate gastric vagal afferents that activate magnocellular and parvocellular oxytocin neurones^3^, ^4^ via a relay in the nucleus tractus solitarii,^5^ and gastric distension may activate the same pathway. Hormonal signals may also affect oxytocin neurones either directly (if they can penetrate the blood–brain barrier), or indirectly via their actions at any of the brain's circumventricular organs, several of which project densely to the magnocellular nuclei. For example, leptin^6^ and secretin^7^ are appetite‐suppressing hormones that activate oxytocin neurones when given systemically, but whether their actions are direct or indirect is not established.

In 2017, Hume et al.^8^ described how gavage of sweetened condensed milk activated oxytocin neurones of the supraoptic nucleus, reporting that this activation could not be accounted for as a response to gastric distension. By contrast, gavage of isocaloric cream of similar viscosity had no significant effect on oxytocin neurones. As gavage of sweetened condensed milk (but not cream) increases the plasma concentration of insulin, we tested the effect of systemic injection of insulin.^9^ This also activated oxytocin neurones, and this was not a consequence of a reduction in plasma glucose because clamping systemic glucose levels did not affect the response of oxytocin neurones. Rather it appeared to be an effect of insulin itself in the brain. Zhang et al.^10^ reported that centrally administered insulin activated oxytocin neurones, and we found that the effects of systemically applied insulin on oxytocin neurones could be blocked by a specific insulin antagonist administered via intracerebroventricular (ICV) injection. The same antagonist also blocked the response of oxytocin neurones to gavage of sweetened condensed milk. Thus, when ingestion of food stimulates insulin secretion, the secreted insulin acts on the brain to stimulate the secretion of oxytocin into the blood and presumably also within the brain.^11^

These experiments involved recording from magnocellular oxytocin neurones in anaesthetised rats. We recently published a computational model that enables predictions of plasma oxytocin concentrations to be made from electrophysiological data of the activity of oxytocin neurones,^12^, ^13^ so measuring oxytocin in response to gavage of sweetened condensed milk would both be an independent test of the conclusions from the electrophysiological studies and a test of the accuracy of predictions made from the computational model.

However, first it was necessary to have an assay which could accurately measure oxytocin in small samples of rat plasma. By the end of the 1980s, many laboratories had developed sensitive and specific radioimmunoassays to measure oxytocin.^14^ The steps involved in validating these assays led to the recognition that most antibodies encountered plasma matrix interference that gave rise to spuriously high measurements in assays of raw plasma. This problem could be circumvented by processing plasma samples to eliminate (“extract”) elements with a high molecular weight, but relatively large sample volumes were generally needed, limiting the number of samples that could be taken from a single rat. However, the problem of matrix interference is specific to species and antibody, and one assay, developed by Higuchi et al.,^15^ used a polyclonal antibody that incurred extensive plasma matrix interference in human plasma but which fortuitously incurred no such interference when used in rat plasma. This assay came to be extensively used in studies in rats, enabling as it did reliable measurements in repeated small blood samples. Unfortunately, stocks of the Higuchi antibody, like those of many other carefully validated antibodies used for oxytocin assays in former years, are now exhausted.

Then, in 2016, Minhas et al.^16^ reported that one commercially available RIA could accurately measure oxytocin in rat plasma without extraction. They reported that the assay was highly sensitive (2.4 pg/mL sample) and that measured oxytocin levels were unaffected by extraction. The levels that they measured in trunk blood from decapitated rats were in the range expected from previous studies in conscious rats (basal <5 pg/mL in males, <20 pg/mL in females) and, as expected, measured levels rose in response to acute stress.

Here, we tested the accuracy and reliability of the assay used by Minhas et al. We then used this assay to measure oxytocin secretion in rats in response to insulin injection and in response to gavage of sweetened condensed milk, and compared the results with values predicted from electrophysiological data using our computational model.

## 2 METHODS

We used adult male Sprague–Dawley rats weighing 300–350 g. The rats had ad libitum access to food and water and were maintained under a 12:12 h light/dark cycle (lights on 7:00 a.m.) at a room temperature of 20–21°C. All procedures were conducted on rats under deep terminal anaesthesia in accordance with the UK Home Office Animals Scientific Procedures Act 1986 and a project licence approved by the Ethical Committee of the University of Edinburgh. Anaesthesia was induced using isoflurane inhalation followed by an intraperitoneal injection of urethane (ethyl carbamate 25% solution) at 1.3 g/kg, and a femoral vein was cannulated for withdrawing blood samples.

### 2.1 Hypophysectomised rat plasma

To collect plasma from hypophysectomised rats, anaesthetised rats were tracheotomised and placed supine in a stereotaxic frame. The pituitary was exposed by transpharyngeal surgery in preparation for its subsequent excision, and a blood sample was withdrawn for measurement of basal oxytocin concentrations. All blood samples were collected in prechilled EDTA‐coated tubes (Microvette CB 300 K2E, Sarstedt) and centrifuged at 630g, for 15 min at 4°C. The pipetted plasma, in 100‐μL aliquots, was placed into 0.5‐ml Eppendorf tubes and stored at −80°C. In some rats, an intraperitoneal (i.p) injection of 1 mL 1.5 M NaCl was given to raise oxytocin concentrations to supraphysiological levels; in these cases, a second blood sample was taken 30 min after the i.p injection. The pituitary was then aspirated under visual control, taking care to confirm directly that the sella turcica was completely emptied of tissue. Two hours later, the rats were exsanguinated, and the plasma separated by centrifugation and stored.

### 2.2 Gavage

Rats (*n* = 18) were fasted overnight (for 16 h) to empty the stomach and to reduce the variability of blood glucose and gastric signals induced by prior food consumption. Rats were anaesthetised as above, and a femoral artery was cannulated for blood sampling. The cannula was flushed, after surgery and between samples, with heparinised saline (50 U/mL). Rats were tracheotomised, and a plastic gavage tube (FTP‐13‐150; Solomon Scientific) was inserted via the mouth into the stomach. In one group of six rats, this was used to deliver 5 mL of sweetened condensed milk (SCM; 40.8 kilojoule [kJ], 0.24 g fat, 1.68 g sugar; Nestle) diluted 50% v/v in distilled water. Another group of six rats were given a gavage of 5 mL of isocaloric cream (fresh single cream), (40.6 kJ, 0.955 g fat, 0.11 g sugar; Tesco UK). A third group of six rats (controls) had a gavage tube inserted but were given no gavage. One hour after inserting the gavage tube, the first blood sample was withdrawn and the gavage was started at 140 μL/min to deliver the 5 mL of SCM or cream over 35 min. Subsequent samples were taken 20 and 40 min after the start of the gavage.

### 2.3 Insulin

In another group of eight rats, anaesthetised as above but without tracheal cannulation or a gavage tube, blood samples were taken before and after intravenous (i.v.) injection of insulin to measure plasma concentrations of oxytocin and glucose. Human recombinant insulin solution (cat no. I9278; Sigma‐Aldrich Company Ltd.) was diluted in 0.9% saline (B. Braun) at 0.25 U/100 μL and injected i.v. at a dose of 0.75 U/kg bodyweight. One sample (0.6.ml) was taken before insulin injection, and two further samples were taken at 30 and 60 min after injection. Blood glucose concentrations were measured in 50 μL of plasma using an Accu‐Chek Aviva meter (Roche Diagnostics GmbH); oxytocin was measured in duplicate in 25‐μL aliquots of plasma.

The dose of insulin given was the same as used by Paiva and Leng,^9^ which had been selected as a commonly used “low” dose of insulin, apparently commonly designated as such for its moderate effect on plasma glucose concentrations. In the study of Paiva and Leng, this dose reduced plasma glucose concentrations by about 50% over the following 60 min.

### 2.4 Radioimmunoassay

**FIGURE 1:**
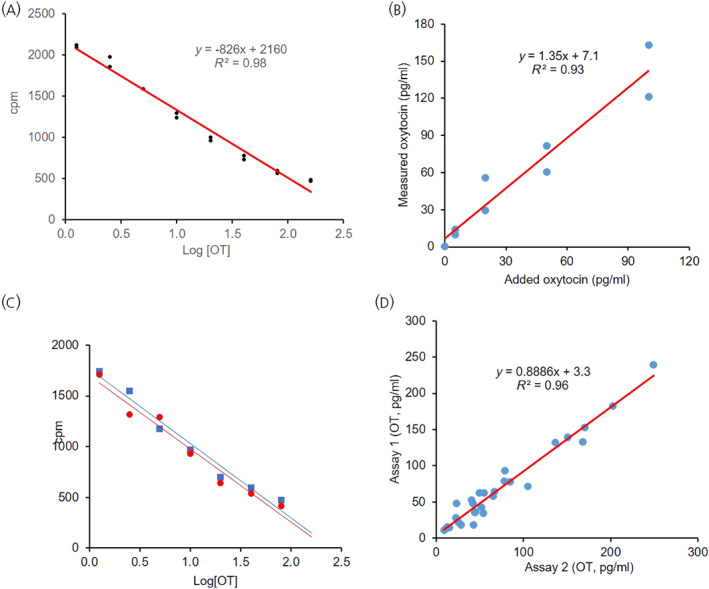
(A) Representative standard curve, plotting the log of oxytocin concentration against measured counts/min (cpm). The standards were constructed by adding dilutions of a stock solution of oxytocin to buffer volumes of 100 μL to give concentrations between 1.25 and 160 pg/mL oxytocin. The points show the duplicate measurements, the red line is the best fit to the mean of the duplicates. Note that the curve is linear through the lowest concentration of oxytocin. (B) Here we measured in duplicate, 100 μL samples of hypophysectomised rat plasma to which oxytocin had been added to give final concentrations of 5, 20, 50 or 100 pg/mL. Oxytocin was undetectable in samples to which no oxytocin was added (in this assay the nominal value measured was 0.6 pg/mL, below the lowest standard (1.25 pg/mL). The measured concentrations of oxytocin in the other samples exceeded the expected true concentration by a factor of 1.3 as shown by the slope of the fitted line (red), indicating some plasma matrix interference for 100‐μL sample volumes. (C) In another assay, a standard curve in buffer (red) was constructed as in A and is compared with a curve where for each standard, before the addition of oxytocin, 25 μL of buffer was replaced by 25 μL of plasma from a hypophysectomised rat. The standard curve (blue) is closely superimposable on the standard curve constructed in buffer alone, indicating that there is no interference from the plasma matrix when sample volumes of 25‐μL are used. (D) Here 25‐μL aliquots of the same 38 plasma samples were measured in duplicate in two successive assays using different kits, 1 month apart. The means of the duplicates are compared for the two assays and the trend line of best fit is shown. The good fit and concordance of measurements in the two assays indicates strong interassay consistency.

Oxytocin measurements were made using a ^125^I kit (Phoenix Pharmaceuticals); according to the manufacturers, the antibody does not cross‐react with vasopressin. We followed the methodology of Minhas et al.^16^ closely. Specifically, plasma samples were not extracted. After exploratory studies to evaluate plasma matrix interference, including some described in the Results, samples were measured in duplicate with a plasma volume of 25 μL. Assay standard curves were linear throughout the range (mean *R* ^2^ values for 6 assays 0.97 ± 0.01, range 0.93 to 0.98) (Figure 1A). The intra‐assay coefficient of variation, estimated in six assays from the variance of counts between sample duplicates, was 6.2 ± 0.8% (range 4.9% to 9.2%) (Figure 1B). The interassay coefficient of variation, estimated from repeated measurements of plasma samples in successive assays 4–6 weeks apart, was 10 ***±*** 2%.

### 2.5 Electrophysiological data

We obtained the electrophysiological records of oxytocin cells recorded during gavage of sweetened condensed milk in the experiments of Hume et al^8^ (9 cells) and Paiva and Leng^9^ (5 cells). These experiments were all performed in urethane‐anaesthetised adult male rats, using a transpharyngeal approach to expose the supraoptic nucleus and neural stalk; and supraoptic neurones were identified as oxytocin neurones by their excitatory response to i.v. injection of cholecystokinin – a stimulus that increases oxytocin secretion but inhibits vasopressin neurones. We characterised the basal firing pattern of each cell as described in detail elsewhere.^17^ For each cell, we selected a stable section of the recording of spontaneous activity before the gavage, and determined the interspike interval (ISI) distribution in 5‐ms bins, and calculated the hazard function according to the formula (hazard in bin [*t*, *t* + 5]) = (number of ISIs in bin [*t*, *t* + 5])/(number of ISIs of length > *t*). A hazard function plots how the excitability of a neurone evolves after a spike has fired and it reflects the superimposed effects of Ca^2+^‐ and voltage‐dependent currents that are triggered by a spike, and the perturbations of afferent input that result from that spike. To capture longer timescale patterning we calculated the index of dispersion (IoD) as the variance/mean rate for different bin widths (from 0.5 to 10 s). These statistical parameters were subsequently used for fitting the computational models.

The firing rates for each cell, from 10 min before the onset of gavage to 35 min after the onset, were calculated as the number of spikes in each 30‐s bin. The magnitude of the response to gavage was not significantly related to the initial firing rate (*R* ^2^ = 0.01) and so we normalised these values by subtracting the average number of spikes/bin in the 10‐min period before onset of gavage.

### 2.6 Computational modelling

We used our published model of oxytocin neurones^17^ modified by the addition of a fast depolarising after potential (DAP), described in our model of vasopressin neurones.^18^ The model implements the equation

$$V=V_{\text{rest}}+V_{\mathrm{syn}}-\mathrm{HAP}-\mathrm{AHP}+\mathrm{DAP}$$

where *V* is the membrane potential, *V*_rest_ is the resting potential, and *V*_syn_ is the summed synaptic input signal, consisting of randomly timed perturbations simulating a mixture of excitatory and inhibitory postsynaptic potentials. After each spike, the neuronal excitability is modulated by a hyperpolarising afterpotential (HAP), a fast depolarising afterpotential (DAP), and a medium afterhyperpolarisation (AHP). Each of these is modelled using a single variable that is step incremented with each spike and decays exponentially, defined by a magnitude (*k*) and a decay rate (or half‐life) parameter (λ). In the model excitatory and inhibitory postsynaptic potentials arrive randomly at the same mean rate (*I*_re_), with a fixed amplitude of 2 mV and a fixed half‐life of 7.5 ms. The cells have a resting potential of −62 mV and a spike threshold of −50 mV; these parameters were kept constant for all model cells.

To fit the model to the spike rate and patterning data from the 13 oxytocin neurones, we used an automated fitting procedure based on a genetic algorithm (GA). The procedure is described in detail in Leng et al.^19^ The fitting process results in a quantitative measure of goodness–of‐fit for every parameter set used, calculated as the weighted sum of root mean square error measures comparing the ISI histogram, hazard function, and IoD range between recorded and model generated spike times: a smaller value indicates a better fit. The fitting varied seven parameters, defining the synaptic input rate, and the three pairs of *k* and *λ* for the HAP, AHP, and DAP. Most oxytocin neurones exhibit an HAP and AHP, but only some show evidence of a DAP. Each cell was fitted with four versions of the spiking model: using only an HAP, HAP + AHP, HAP + DAP, and HAP + AHP + DAP. The best fit was selected from 100 runs of the GA with each of the four models. From these 13 fits we constructed a “typical oxytocin cell” parameter set as given in Table 1.

**TABLE 1:** Spiking model best fits to recorded oxytocin neurones.

Cell	*I*_re_	*k*_HAP_	*λ*_HAP_	*k*_DAP_	λ_DAP_	*k*_AHP_	*λ*_AHP_
n130211	375	48.39	6.1			0.34	545
n130212 − 2	484	63.47	5.2	4.30	21.0	0.09	1329
n130214‐2	486	48.35	6.8	4.10	28.2	0.58	468
n130215	350	30.26	7.5	5.00	31.3	0.31	337
n140924	281	10.11	15.9				
n141002	341	88.51	5.4	0.60	61.3		
n141021‐2	466	39.62	6.5	5.88	37.3	1.13	482
n141022‐5	472	44.16	5.2	3.77	20.1	1.01	357
LP181212	268	19.61	11.9	4.14	43.1		
LP181219	357	51.93	7.2			0.39	702
LP181220	367	58.16	7.7			1.08	632
LP181221	383	48.75	9.7	3.24	74.1	0.61	659
LP190109	360	11.64	15.6			0.24	561
							

HAP only	HAP + DAP	HAP + AHP	ALL				

*Note*: Cell names beginning “n” are from the study of Hume et al.,^8^ those beginning LP are from Paiva and Leng.^9^

Abbreviations: AHP, afterhyperpolarisation; DAP, depolarising afterpotential; HAP, hyperpolarising afterpotential.

We then used the model of Maicos‐Roya et al.^12^ to predict plasma oxytocin concentrations from the average electrophysiological response. That model extends the spiking model with a secretion model, fit to data on stimulus‐secretion coupling in the posterior pituitary; and a model that mimics the plasma diffusion and clearance of oxytocin in rats with a two‐compartment model, replicating the dynamics observed experimentally after infusion and injection of oxytocin.

## 3 IMPLEMENTATION

The model was developed and run using our own HypoMod software https://github.com/HypoModel/MagNet written in C++ with a graphical interface based on the open‐source wxWidgets library. The software simulates a population of oxytocin neurones by running multiple threads in parallel, summing the secretory output of the neurones to drive a single thread running the model of plasma oxytocin concentration. A single run using a 1‐ms step size to simulate 6000 s of activity of 100 neurones responding to a gavage protocol takes 18 s, running on an eight core AMD Ryzen 75800X 4.2GHz processor.

## 4 RESULTS

### 4.1 Assay validation

We established that 100 μL of plasma from hypophysectomised rats contained no oxytocin detectable by the assay (<1.25 pg/mL), whereas plasma samples from urethane‐anaesthetised pituitary intact rats taken 40 min after i.p injection of hypertonic saline by cardiac puncture indicated, as expected, a very high concentration of oxytocin (levels above the top standard, >160 pg/mL). When 1‐mL aliquots of plasma from hypophysectomised rats were spiked with 5, 20 and 50 and 100 pg oxytocin, the oxytocin measured in 100‐μL samples increased linearly with concentration with a gain of 1.3, indicating a modest level of plasma matrix interference, consistent with some occlusion of oxytocin binding to the oxytocin antibody (Figure 1C). Aliquots of the same samples were reassayed in a subsequent assay with similar results. In subsequent investigations we reduced the sample volume to 50 and 25 μL.

In one assay we constructed two standard curves, one in normal assay buffer and one in which, in every tube, 25 μL of buffer was replaced by 25 μL of hypophysectomised rat plasma (Figure 1D). In another assay we similarly constructed two standard curves, this time replacing 50 μL of buffer with 50 μL of hypophysectomised rat plasma. In each case, the two standard curves fully superimposed, indicating that with sample volumes of 25 or 50 μL there was no detectable plasma matrix interference. We therefore concluded that the assay would accurately measure oxytocin in 25‐μL samples of plasma. Standard curves were consistently linear throughout the range of standards (corresponding to 5–640 pg/mL plasma for 25‐μL samples; Figure 1A). Minhas et al. reported that standard curves maintained linearity when an additional low standard was included (corresponding to 2.5 pg/mL), but as levels in urethane‐anaesthetised rats are relatively high, we saw no need for this in our experiments. We used this assay to measure oxytocin secretion in response to intravenous injection of insulin, and to gavage of sweetened condensed milk or cream.

Paiva and Leng^9^ reported that in urethane‐anaesthetised male rats, i.v. injection of 0.75 IU insulin increased the firing rate of oxytocin neurones by a mean of 1.6 spikes/s in the 30 min after injection, a rate which was sustained for at least another 30 min. Consistent with this, we observed here that, in eight rats injected with the same dose of insulin, the mean (SEM) basal oxytocin concentration of 80 ± 18 pg/mL increased by 63 ± 20 to 142 ± 34 pg/mL at 30 min after injection (*p* = .015; paired *t* test) and this elevated level was unchanged at 60 min (149 ± 32 pg/mL). Plasma glucose concentrations fell from 9 ± 0.8 to 4.8 ± 0.4 mM at 30 min and 4 ± 0.3 mM at 60 min, a change very similar to that found by Paiva and Leng (2020).

Hume et al.^8^ reported that gavage of sweetened condensed milk activated oxytocin neurones while gavage of isocaloric cream had no significant effect. In six rats given a sham gavage, we saw no significant change in plasma oxytocin concentration (46 ± 14 pg/mL at basal, 48 ± 17 pg/mL at 20 min and 64 ± 20 pg/mL at 40 min, for a mean change of 18 ± 10 pg/mL). By contrast, in six rats given sweetened condensed milk, levels of oxytocin rose from a mean of 65 ± 22 pg/mL at basal to 105 ± 27 pg/mL at 20 min, and in every rat they rose again to 229 ± 56 pg/mL at 40 min for a mean change of 164 ± 40 pg/mL. In six rats given isocaloric cream, levels of oxytocin rose (non‐significantly) from 49 ± 7 pg/mL to 84 ± 16 pg/mL at 20 min, and levels at 40 min showed no further change, at 86 ± 8 pg/mL, for a mean change of 37 ± 5 pg/mL. The differences at 40 min were compared by one‐way ANOVA (*F*‐ratio 8.2, *p* = .004); Tukey's HSD procedure determined that the difference between the sweetened condensed milk group and the cream group was significant at *p* = .014, and the difference between the sweetened condensed milk group and the sham group was significant at *p* = .005. No significant difference was detected between the cream group and the sham group.

### 4.2 Computational modelling

**FIGURE 2:**
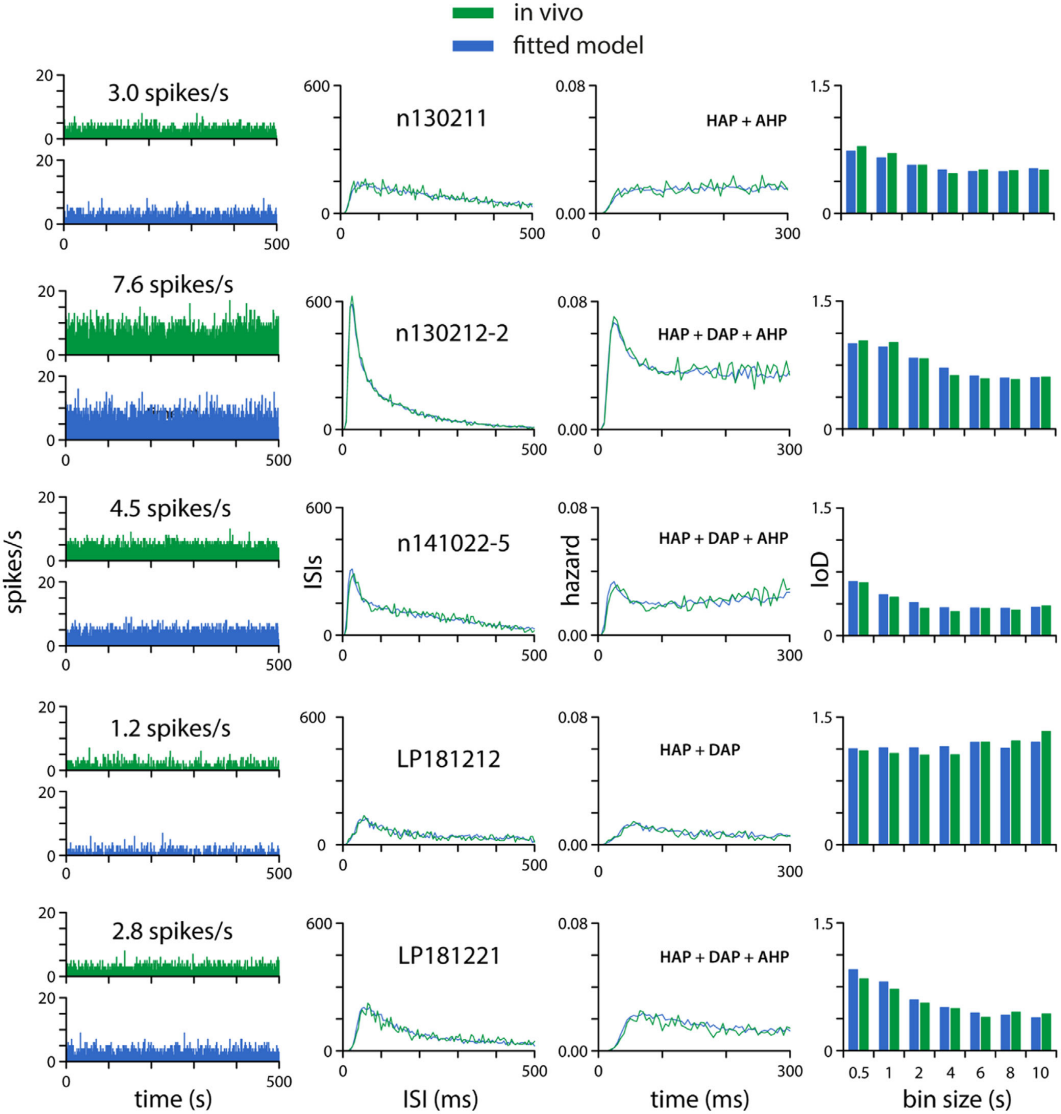
Spiking model fits to in vivo recorded oxytocin neurones Using genetic algorithm based automated fitting, we produced close matches to the spike patterning of 13 neurones, using spike times from a ~30 min stable period recorded before the gavage stimulus was applied, matched by an integrate‐and‐fire based model receiving random synaptic input at a fixed rate. This figure shows five representative neurones. Each row shows a single neurone and its best model fit. The first column shows a 500‐s recording of spontaneous firing rate in 1‐s bins for the recorded cell (green) and the matching model cell (blue). The second column shows superimposed ISI histograms (in 1‐ms bins) for the recorded cell and model cell; the third column shows superimposed hazard functions (in 5‐ms bins) and the fourth column compares IoD values for bins between 0.5 and 10 s. The goodness of fit of the model is calculated as a weighted sum of the match to the ISI distribution, the hazard function, and the IoD range.

We combined the electrophysiological records of the nine oxytocin cells recorded during gavage of sweetened condensed milk in the experiments of Hume et al.^8^ with records of the five cells reported by Paiva and Leng.^9^ The 14 oxytocin cells had a mean basal firing rate of 3.26 ± 0.8 spikes/s (median 3.1 spikes/s, range 1.3–6.6 spikes/s). We fitted a spiking model to 13 of the oxytocin cells using a GA‐based automated fitting procedure to match baseline measures of spike activity and patterning; the other cells did not have a sufficiently long period of stable baseline activity to allow a good fit. The best fit parameters and fit scores are given in Table 2. Figure 2 shows five of the 13 fitted cells, showing the fit to spike rate, ISI histogram, hazard function, and IoD range.

**TABLE 2:** Fit derived standard spiking model parameters.

Name	Description	Value (units)
*I*_re_	Excitatory input rate	370 (Hz)
*I*_ratio_	Inhibitory input ratio	0.5
*e*_h_	EPSP amplitude	2 (mV)
*i*_h_	IPSP amplitude	−2 (mV)
*λ*_syn_	PSP half life	7.5 (ms)
*k*_HAP_	HAP amplitude per spike	50 (mV)
*λ*_HAP_	HAP half life	8 (ms)
*k*_DAP_	Fast DAP amplitude per spike	3 (mV)
*λ*_DAP_	Fast DAP half life	50 (ms)
*k*_AHP_	Medium AHP amplitude per spike	0.5 (mV
*λ*_AHP_	Medium AHP half life	600 (ms)
*V*_rest_	Resting potential	−62 (mV)
*V*_thresh_	Spike threshold potential	−50 (mV)

Abbreviations: AHP, afterhyperpolarisation; DAP, depolarising afterpotential; EPSP, excitatory postsynaptic potential; HAP, hyperpolarising afterpotential; IPSP, inhibitory postsynaptic potential; PSP, postsynaptic potential.

We used these 13 fits to construct a “consensus” oxytocin cell parameter set. Of the 13 cells, one could be closely fit using a HAP alone, four with a HAP and an AHP, one with a HAP and a DAP, and six needed all three. For the HAP there was a negative correlation (*R* ^2^ = 0.64) between the amplitude (*ƙ*_HAP_) and the time constant (*λ*_HAP_), and the values 50 mV and 8 ms, respectively were selected as a point lying on the line of best fit close to the median and mean values of each. Ten of the cells required an AHP for the best fit, and we chose rounded median values of 0.5 mV and 600 ms for *ƙ*_AHP_ and *λ*_AHP_. There was no evidence of a relationship between the AHP parameters, but as for the HAP parameters there was an inverse correlation between *ƙ*_DAP_ and *λ*_DAP_. We noted that there is also evidence of a negative correlation between the amplitude and the time constant for the DAP and AHP, but as stated these were not present in all of the fits.

**FIGURE 3:**
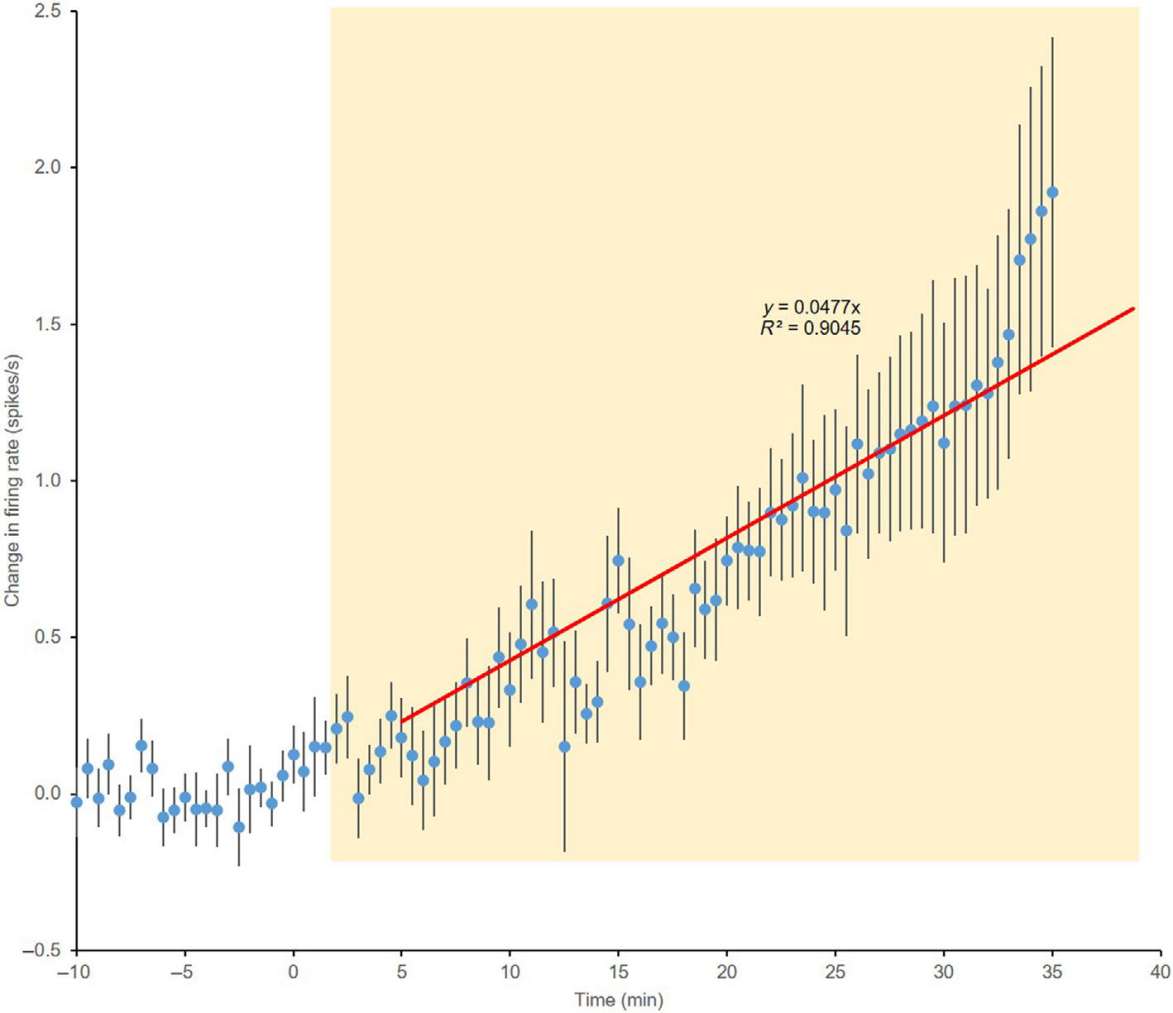
Response of oxytocin neurones to gavage of sweetened condensed milk. Data from 14 oxytocin neurones recorded from the supraoptic nucleus of urethane‐anaesthetised rats. The figure shows their response to gavage of sweetened condensed milk (during the period indicated by the yellow shaded panel). Data are spike rates in 30‐s bins, normalised for each cell to the average rate in the 10‐min before beginning the gavage. The data points are thus mean changes from basal firing rate ± SEM. The red trendline is the linear fit to data from 3.5 min after the start of the gavage.

We then characterised the change in activity during gavage. One cell was lost after 15 min of gavage, but the other 13 were recorded for at least 33.5 min. In these cells, the mean firing rate rose during the gavage, approximately linearly, by 1.5 ± 0.4 spikes/s at 30–35 min (Figure 3). The response magnitude was independent of the initial firing rate, but there appeared to be a variable delay between the beginning of gavage and the onset of a response. The first bin after which the mean change in firing rate was consistently greater than 0 was from 3.5 to 4 min after the start of the gavage, so we fit a straight line to the mean data from the 14 cells from this point to 35 min after the start, assuming an intercept at +3.5 min. The best fit line had a slope of 0.477 (*R* ^2^ = 0.91) (Figure 2).

**FIGURE 4:**
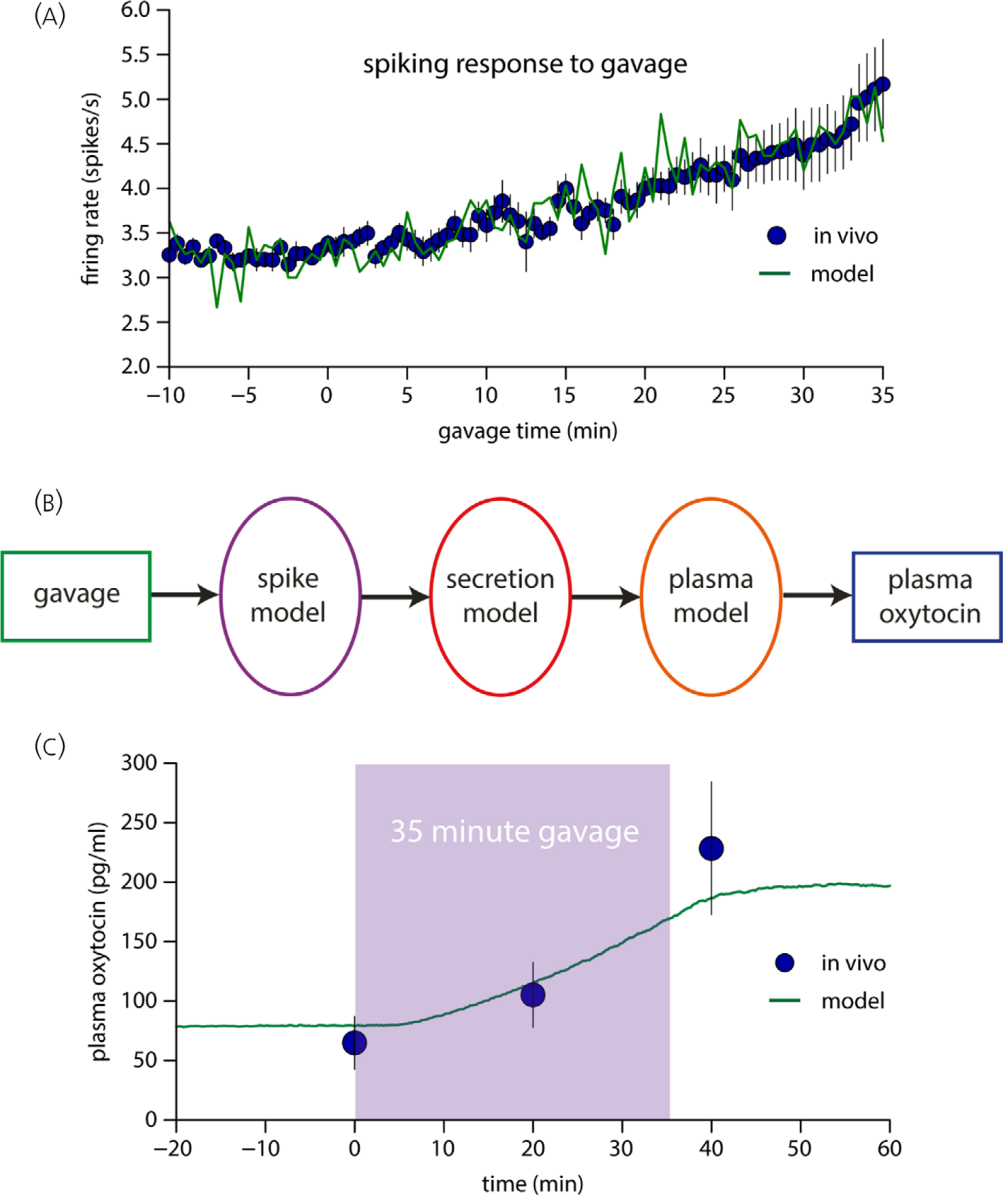
Coupled spiking, secretion, and plasma model used to simulate plasma oxytocin response to gavage (A) shows the firing rate of a model oxytocin neurone (green) during simulated gavage, superimposed on the observed average firing rate of 14 oxytocin neurones recorded gavage. (B) A schematic of the coupled model, simulating the full path input stimulus to output plasma oxytocin signal. (C) Shows the model prediction of plasma oxytocin concentrations during gavage, and the measured mean (SEM) oxytocin concentrations in such experiments.

We then used this relationship to predict the plasma oxytocin concentration in urethane‐anaesthetised rats during gavage by coupling the “consensus” oxytocin spiking model to the secretion and plasma model of Maicas‐Royo et al.^12^ We ran the spiking model coupled to the secretion model 100 times with independent, randomly‐generated synaptic input signals arriving at the same mean rate *I*_re_ (Figure 4). The 100 secretion signals were then averaged to provide a smoothed input to the plasma model to produce a smooth predicted plasma concentration. This was then multiplied by the scaling factor, defined previously,^12^ to relate the output of a single neurone to that of the whole population. That factor had been defined by comparing the model output with plasma concentrations measured in a range of experiments in rats using the Higuchi assay.

We adjusted the mean synaptic input rate *I*_re_ to 370 Hz to give a basal firing rate that closely matched the observed rate of 3.26 spikes/s. We maintained this input for a 40‐min control period and for a further 3.5 min after the start of the simulated gavage, and then increased *I*_re_ by 0.037 Hz/s for 35 min, followed by 20 min of synaptic input at the post‐ramp elevated mean rate of 448 Hz. The results are illustrated in Figure 4C, overlain on the plasma measurements described above. The model was thus fitted purely on the basis of the electrophysiological data, but shows a close qualitative and quantitative match to the mean oxytocin concentrations measured in response to the same gavage protocol.

## 5 DISCUSSION

Here, we confirmed that, as reported by Minhas et al., the Phoenix RIA kit can sensitively measure oxytocin in unextracted samples of rat plasma. With sample volumes of 25 μL plasma, there appears to be no plasma matrix interference, as indicated by the finding that this volume of plasma from hypophysectomised rats had no observed effect when substituted for the same volume of assay buffer when constructing the assay standard curve.

Thus, the Phoenix RIA can reliably measure oxytocin in small volumes of unextracted rat plasma, and this is important because of widespread doubts raised about the analytical integrity of many oxytocin assays in current use. Problems with plasma matrix interference are common in immunoassays; they can be circumvented by extracting plasma samples to eliminate the high molecular weight elements that interfere with assay performance,^20^ but this entails larger sample volumes, an additional processing step, and some loss of signal, as between 10% and 40% of the oxytocin in plasma is typically lost during extraction. However, in recent years, many studies have published measurements of oxytocin in unextracted plasma, typically justifying this approach on the basis that others have used it. These studies have reported very high levels of oxytocin that are apparently uncorrelated with true levels of free oxytocin.^14^, ^21^, ^22^, ^23^, ^24^

For example, the Enzo ELISA kit has commonly been used to measure oxytocin in unextracted plasma, although the manufacturers recommend that plasma and serum samples should be extracted before being assayed, and despite the attention that has been paid to this issue.^14^, ^21^, ^22^, ^24^, ^25^, ^26^ For example, one recent study used the Enzo assay to measure oxytocin in unextracted human plasma, reporting mean levels of >1 ng/mL in men and >1.5 ng/mL in women. By contrast, Szulc et al.^27^ used the Phoenix RIA that we used here to measure oxytocin in extracted serum from a cohort of 550 men and reported a median level of 0.74 pg/mL, broadly consistent with most measurements of oxytocin in extracted human plasma by diverse RIAs.

The extent of plasma matrix interference varies between different assay kits,^23^, ^26^, ^28^ and the Enzo assay may have a particularly serious problem with plasma matrix interference: recently it was reported to measure mean levels of 813 pg/mL in plasma from transgenic mice that are completely deficient in oxytocin.^26^ Some other ELISAs, including the Arbor Assays kit, may have less of a problem, at least when used to measure in mouse plasma. A study using plasma from transgenic oxytocin‐deficient mice to identify plasma matrix interference showed that this assay could reliably measure oxytocin in unextracted mouse plasma diluted 1:8 (Gnanadesikan et al., 2021). However, this finding probably does not generalise to all other species; plasma matrix interference varies with species and possibly also from individual to individual within species, and the Arbor Assays kit instructions state that plasma samples should be extracted. The importance of this was demonstrated in a recent study using this assay, which reported mean levels of 186 pg/mL in unextracted samples of human plasma compared to 2.6 pg/mL after extraction.^29^ There was a positive correlation between the measurements, but the relative weakness of the correlation suggested interference with some factor that varied between individuals. Accordingly, while the present study validates the use of the Phoenix RIA for measuring oxytocin in unextracted rat plasma, this gives no good grounds for expecting it to be similarly useful in other species.

The experimental results in the present paper show that, in rats, gavage of sweetened condensed milk and i.v. injections of insulin both stimulate oxytocin secretion. Thus, we have confirmed, by an independent technical approach, conclusions drawn previously from electrophysiological studies and studies of c‐*fos* expression.^8^, ^9^ The potential significance of this finding lies in the current interest in oxytocin as a possible treatment for reducing obesity and ameliorating diabetes.^30^, ^31^, ^32^ Clearly oxytocin has diverse actions both centrally and peripherally that influence many metabolic parameters, including food intake, but we remain far from understanding its physiological regulation in this context.

The central release of oxytocin appears to modulate the motivation to eat by its actions not only in the hypothalamus, but also in the amygdala^33^ and at forebrain sites such as the nucleus accumbens^2^, ^34^, ^35^, ^36^ and the ventral tegmental area,^37^, ^38^ where it may be involved in food reward. There is also evidence that oxytocin can suppress food intake by its peripheral actions.^31^, ^39^

The functional organisation of central projections of magnocellular oxytocin neurones, and the relative contribution of axons and dendrites to central oxytocin release, have yet to be fully defined, despite its potential importance for understanding central oxytocin signalling. Whereas classical tracing studies revealed little indication that magnocellular neurones projected anywhere except to the posterior pituitary, recent studies have indicated that a minority of the magnocellular neurones project axon collaterals to diverse forebrain sites. For example, in prairie voles, Ross et al.^40^ demonstrated by retrograde tracing that the nucleus accumbens receives oxytocin fibres from a subpopulation of magnocellular neurones in the supraoptic and paraventricular nuclei, although neither of these sites had been reported as labelled after injection of retrograde tracers into the rat nucleus accumbens.^41^, ^42^ In mice, Choe et al.^43^ used viral anterograde tracing to show that oxytocin fibres in the piriform cortex arise from both the supraoptic and paraventricular nuclei, and in the rat, Zhang et al.^44^ expressed viral tracers in neurones that projected to the posterior pituitary in rats to show that magnocellular neurones project to a wide range of forebrain sites, including the piriform cortex, nucleus accumbens and amygdala (but not to the ventral tegmental area). In mice, the ventral tegmental area has been reported to contain oxytocin fibres that arise from the paraventricular nucleus (but not from the supraoptic nucleus). Thus parvocellular oxytocin neurones might project to more sites than the acknowledged major targets in the dorsal vagal complex and spinal cord, but, given the difficulty of selectively labelling the parvocellular neurones, and the possibility of species differences in central oxytocin projections the organisation of central oxytocin projections remain uncertain.

Why these “new” projection sites were not reported previously by the many classical studies using retrograde tracers is not clear. The main route by which retrograde tracers are taken up by neurones is thought to be in the process of endocytosis by which membrane is recovered after exocytosis. If these axon collaterals contain (and release) few oxytocin‐containing vesicles, there may be little uptake of retrograde markers, at least compared to the primary axon terminal fields of the posterior pituitary. As magnocellular oxytocin neurones have a glutaminergic phenotype it may be that glutamate is the important messenger of these collateral branches, and this may only occur at classical synapses. However, even a small amount of oxytocin release from axonal varicosities might be powerfully effective. As we have noted elsewhere, compared to conventional synaptic vesicles, oxytocin‐containing vesicles carry a much larger cargo, and one that is far more potent (by acting at receptors with nanomolar affinity), and far more enduring (as oxytocin has a half‐life measured in minutes rather than milliseconds).^45^

While, as we show here, insulin secretion stimulates oxytocin secretion in rats, oxytocin released within the mouse brain by a subset of parvocellular oxytocin neurones can inhibit insulin secretion by an action involving the sympathetic autonomic innervation of pancreatic β cells.^46^ This may indicate a homeostatic feedback loop in the regulation of glucose homeostasis, but this hypothesis presumes that parvocellular and magnocellular oxytocin neurones are a functionally coherent unit, and the apparent presence of both oxytocin and functional oxytocin receptors in the pancreas^47^, ^48^, ^49^ raises further important question of the relationship between the enteric oxytocin system and the hypothalamo‐neurohypophysial system. It seems likely that the ability to measure oxytocin secretion reliably in rodents will be important in addressing this. We found that the apparent limit of detection of plasma oxytocin was about 5 pg/mL with 25 μL sample volumes, but there was no apparent plasma matrix interference with 50 μL sample volumes, so we expect that the assay could be used to measure plasma concentrations as low as 2.5 pg/mL ‐ about the basal concentration generally measured in conscious rats. Here we used urethane anaesthetised rats, where plasma concentrations are much higher and very comfortably in the measurable range.

The plasma oxytocin levels during gavage were very close to the levels predicted from a computational model, which predicted the plasma changes in oxytocin from the electrophysiological responses of oxytocin neurones to gavage. The model itself, as described fully in earlier publications, was built using published data on stimulus‐secretion coupling, plasma clearance of oxytocin, and calibrated using published plasma levels measured using the Higuchi assay.

The match between measured oxytocin levels and model predictions appears to be remarkably close at all three measurement points, and we must therefore emphasise that the model was not fitted to the oxytocin data in any respect – it was fitted solely to published electrophysiological data using a previously model of stimulus‐secretion coupling and plasma clearance that was developed using older published data. In other words, this is a demonstration of the quantitative predictive accuracy of the models involved.

This is not of course to say that the models are as good as they might be: they involve many simplifications, and have considerable scope for refinement; however, the intrinsic variability in experimental measurements of plasma oxytocin may mean that the superiority of a more refined model is unlikely to be demonstrable by such comparisons. The next phase in model evolution should comprise investigating the model behaviour in silico in order to identify unexpected or counter‐intuitive predictions that might offer a rigorous and demanding test of the model.

## AUTHOR CONTRIBUTIONS

**Shereen Hassan:** Formal analysis; investigation; methodology; writing – review and editing. **Hala El Baradey:** Funding acquisition; supervision. **Mohamed Madi:** Supervision. **Mohamed Shebl:** Supervision. **Gareth Leng:** Conceptualization; data curation; formal analysis; funding acquisition; methodology; project administration; supervision; writing – original draft. **Maja Lozic:** Investigation; methodology; writing – review and editing. **Mike Ludwig:** Conceptualization; funding acquisition; methodology; project administration; writing – review and editing. **John Menzies:** Funding acquisition; methodology; project administration; supervision; writing – review and editing. **Duncan James MacGregor:** Conceptualization; data curation; formal analysis; funding acquisition; investigation; methodology; project administration; software; validation; visualization; writing – original draft; writing – review and editing.

### PEER REVIEW

The peer review history for this article is available at https://www.webofscience.com/api/gateway/wos/peer-review/10.1111/jne.13303.

## Data Availability

The data that support the findings of this study are available from the corresponding author (duncan.macgregor@ed.ac.uk) upon reasonable request.
